# An Improved BLE Indoor Localization with Kalman-Based Fusion: An Experimental Study

**DOI:** 10.3390/s17050951

**Published:** 2017-04-26

**Authors:** Jenny Röbesaat, Peilin Zhang, Mohamed Abdelaal, Oliver Theel

**Affiliations:** 1 OFFIS—Institut für Informatik, 26121 Oldenburg, Germany; jenny.roebesaat@offis.de; 2 Department of Computer Science, Carl von Ossietzky University of Oldenburg, 26111 Oldenburg, Germany; theel@informatik.uni-oldenburg.de; 3 Institute for Parallel and Distributed Systems, University of Stuttgart, 70569 Stuttgart, Germany; mohamed.abdelaal@ipvs.uni-stuttgart.de

**Keywords:** indoor localization, Bluetooth Low Energy, Kalman filter, dead reckoning, trilateration, data fusion

## Abstract

Indoor positioning has grasped great attention in recent years. A number of efforts have been exerted to achieve high positioning accuracy. However, there exists no technology that proves its efficacy in various situations. In this paper, we propose a novel positioning method based on fusing trilateration and dead reckoning. We employ Kalman filtering as a position fusion algorithm. Moreover, we adopt an Android device with Bluetooth Low Energy modules as the communication platform to avoid excessive energy consumption and to improve the stability of the received signal strength. To further improve the positioning accuracy, we take the environmental context information into account while generating the position fixes. Extensive experiments in a testbed are conducted to examine the performance of three approaches: trilateration, dead reckoning and the fusion method. Additionally, the influence of the knowledge of the environmental context is also examined. Finally, our proposed fusion method outperforms both trilateration and dead reckoning in terms of accuracy: experimental results show that the Kalman-based fusion, for our settings, achieves a positioning accuracy of less than one meter.

## 1. Introduction

The primary goal of indoor positioning systems (IPS) for some users, notably hospitals and malls, is to provide navigation services and tracking solutions. However, others utilize IPS to better market to customers, provide timely information via audio for tours, offer video or augmented reality experiences or connect people of interest in proximity to one another. Similarly, airports can monitor the mobile traffic for crowd control, staff management and alerts, and the airlines can locate passengers, giving them a push notification to start walking towards the gate.

Recently, Microsoft has released a white paper about their indoor localization competition [1]. After analyzing the results of numerous indoor localization solutions, the paper concludes that the indoor localization problem is not solved. There does not exist a technology or a combination of technologies that can recreate the experience that the global positioning system (GPS) offers outdoors in the indoor environment. Furthermore, they confirm that no single solution works perfectly in all environments. Hence, the best solution for indoor positioning might be a hybrid one. These conclusions motivate us to further investigate novel ideas for designing a robust indoor localization method.

Generally, GPS-based localization systems are best suited for outdoor localization. The lack of signal coverage in indoor environments renders GPS not a suitable solution for indoor localization. Hence, several alternative methods were discovered. These methods are based on different information sources like wireless communication technologies or sensor measurements. However, each of them has disadvantages, such as low precision, unreliability, high complexity or high hardware cost. The accuracy problem becomes severe in the case of small-scale indoor buildings. Moreover, buildings and their interiors often have different structures, for example executives’ offices typically have different geometric characteristics than secretaries’ offices. Consequently, the dilemma of designing a suitable indoor localization method is relatively troublesome.

As an emerging technology, mobiles devices, such as smartphones and tablets, have been exploited in the contest of indoor localization. This trend is motivated by the statistics, which estimate the contemporary number of smartphone users as approximately 2.08 billion. This number is expected to approach 2.66 billion user in 2019 [2]. A number of communication facilities have been used in indoor localization, such as WiFi and Bluetooth.

WiFi is a wireless local area network technology based on the IEEE 802.11 standard. Through its increasing availability, more and more devices such as personal computers, smartphones, tablets and video games are equipped with WiFi modules. Bluetooth is a wireless communication technology standard based on the IEEE 802.15.1 and operates in the 2.4-GHz frequency ISM band [3]. Recently, a novel communication facility, referred to as BLE, has been released. The main benefit of such Bluetooth Low Energy (BLE) beacons, compared to the classic Bluetooth, is their low energy consumption, low hardware cost and small size [4]. Therefore, it is rapidly exploited in various new devices and applications such as smartphones or tablets. Table 1 summarizes the advantage and disadvantages of the previously-presented communication technologies.

As can be deduced from Table 1, BLE is well suitable for indoor localization relative to WiFi and the classic Bluetooth. Specifically, BLE signals are not influenced that strongly by the environment because of their lower transmission power [5]. Furthermore, BLE adopts a channel hopping mechanism, leading to fewer package collisions. Finally, BLE has a much higher sampling rate, which makes it easier to filter out outliers. These advantages motivate us to exploit BLE beacons for a precise indoor localization.

The main aim of this paper is to design a precise IPS using a set of pre-deployed BLE beacons and smart mobile devices (e.g., smartphones, tablets and smart watches). In this work, numerous experimental studies of the currently-available indoor localization technologies, including trilateration and dead reckoning, are performed. The primary objective is to examine the level of accuracy and the impact of obstacles and the direction of the mobile device antenna. To get an even higher accuracy, a novel hybrid method, which integrates existing indoor localization methods, namely dead reckoning and trilateration, is investigated. Such an integration aims at emphasizing the advantages of individual methods and relieving their weaknesses. To summarize, the paper has three contributions as follows.

- We perform an extensive experimental study to investigate a BLE-based trilateration method for indoor localization. The study comprises analyzing the BLE received signal strength indication (RSSI) measurements, adopting Kalman filtering to purify the RSSI measurements and eventually estimating the influences of obstacles and antenna’s direction on the collected RSSI measurements.
- Extensive experiments are carried out to study a dead reckoning method for indoor localization. Since dead reckoning relies on sensors readings, we started by analyzing the noise associated with the sensors readings. Additionally, we study several relevant factors such as step length, step counting, heading direction and the characteristics of a human gait.
- A hybrid indoor localization method is proposed using BLE-integrated smartphones to overcome the limitations of both trilateration and dead reckoning. Moreover, a Kalman filter is exploited for the fusion of both methods. We further improve the positioning accuracy via considering the environmental context information such as the building exterior’s width and length. After experimentally examining the proposed fusion method, the obtained results show high accuracy of less than one meter (m). Furthermore, the proposed method is found more robust and reliable relative to the trilateration and the dead reckoning methods. This fact emerges because even if one part of the position estimation mechanism (i.e., trilateration or dead reckoning) fails, the position fortunately can be estimated by the other part.

The remainder of this paper is organized as follows. Section 2 discusses the related work, with a focus on wireless sensor network (WSN)-based and smartphone-based localization approaches. Section 3 defines the research problem and explains the overview of our system model. Section 4 details the trilateration method and examines the RSSI-based localization technique. Section 5 investigates the dead reckoning method, followed by the details of the proposed hybrid localization scheme with Kalman-based fusion in Section 6. Section 7 elaborates the performance evaluation of the various approaches. Finally, Section 8 provides the concluding remarks.

## 2. Related Work

### 2.1. WSN-Based Localization Approaches

During the last decade, a number of efforts has been exerted in developing reliable indoor localization systems. One of these early efforts is the active badge localization system developed by Roy Want et al. in 2001 [6]. In this system, several sensor nodes are deployed at fixed spots inside a building. Users wear an active badge, which transmits a unique code via an infrared sensor every 15 s. The sensor nodes are polled by a location manager software for sightings. This centralized location manager calculates the position of the active badge and then provides location information to the users. Another indoor localization system is the cricket indoor localization system [7]. Cricket uses anchors that transmit ultrasound pulse and radio messages. A mobile node receives these messages and calculates its own position. The results show that positions can be determined with an accuracy of 10 cm. However, these crickets are not practical due to the high hardware cost of the mobile nodes. RADAR [8] is the one of the first indoor localization systems using the WiFi signal. It is based on the fingerprint method, where the reference values are collected during an offline phase and compared with actual measurements during the online phase. The result of the work shows that RADAR localizes user’s laptops with an accuracy of 2 m to 3 m.

### 2.2. Smartphone-Based Localization Approaches

The technical literature comprises many indoor localization systems where users have to wear an additional mobile node. However, these solutions are neither comfortable nor user-friendly since people, in most cases, tend to not wear additional devices. To address this problem, investigations of indoor localization systems with smartphones have had much effort put into them. One of these systems uses several sensors of the smartphone to perform localization in [9]. At the outset, an initial location should be preset before starting the localization algorithm. Then, the smartphone sensors are used to detect steps and the length of a step, as well as the heading direction. The position is then estimated based on the sensor measurements. However, some of the sensors, such as the magnetic sensor for the heading direction, cause interference. Furthermore, the emerging error grows greatly over time, which makes the system unreliable. Another approach is described in [10]. In this approach, the position is calculated according to the RSSI of a WiFi network and the fingerprint method. A major challenge of the fingerprint approach is the large variance of RSSI signals, resulting in low accuracy and low reliability. Besides, another disadvantage of this method is the overhead of utilizing databases, which consumes much time to set up the database, especially for large buildings. Because of the disadvantages of the internal sensor and the fingerprint solutions, [11] presents a hybrid solution that fuses smartphone sensor measurements with WiFi RSSI measurements. In this approach, a filter based on the Markov model is used for the fusion. The results show that the system is able to obtain an accuracy of about 1.5 m.

### 2.3. Discussion

To sum up, the mentioned indoor location systems suffer from several drawbacks. The WSN-based methods have the disadvantage that people have to wear additional hardware, making the solution not user-friendly. On the other hand, the smartphone-based methods are more user-friendly, but using the sensors in smartphones is not reliable because of interference and the growing error. Combining the smartphone and the fingerprint method to determine the location is more precise and reliable, but the effort to set up the required database, especially for large buildings, is huge. Furthermore, the hybrid solution shows an acceptable accuracy, but WiFi might not be as accurate as BLE for RSSI-based measurements. In summary, Table 2 lists the different approaches in terms of the accuracy, advantages and disadvantages.

## 3. Overview

In this section, we define the research problem, and subsequently, we explain the system model.

### 3.1. Problem Formulation

Consider a device user in a building who wishes to navigate through the building interiors. The building is equipped with *m* BLE modules, each of which can be identified by a unique identification (ID) number. Such BLE modules are typically deployed in predefined anchor points. Equation (1) shows the error $e_{ml}$ as the Euclidean distance between the obtained position $p_{ml}$ and the actual position ${\hat{p}}_{o}$, where *i* denotes the exploited position fix. The position $p_{ml}$ refers to the position of a mobile device obtained via adopting the multilateration method. This deviation emerges due to several factors such as RSSI instability, obstacles distraction and multipath fading.

(1) $$e_{ml}=\sqrt{\sum_{i=1}^{m}{\left(p_{ml}\left(i\right)-{\hat{p}}_{o}\right)}^{2}}\;\;\;\;\;\left(i=1,2,\ldots ,m\right)$$

Alternatively, the position fix is feasible without the help of the BLE modules. Dead reckoning typically utilizes a set of embedded sensors at each mobile device to determine the position $p_{dr}$. Specifically, the accelerometer, magnetometer and orientation sensor are involved in the localization process provided that the initial position $p_{init}$ is already known. Equation (2) defines the position estimation using dead reckoning in terms of the initial position $p_{init}$ and the step length $l_{s}$, where *w* is the total number of steps. Since dead reckoning relies on estimating the step length, the Euclidean error $e_{dr}$ continuously grows as given in Equation (3). The dead reckoning position $P_{dr}\left(l_{s},t\right)$ is expressed in terms of the step length and the total number of steps.

(2) $$p_{dr}=p_{init}+l_{s}\;\cdot \;w$$

(3) $$e_{dr}=\sqrt{{\left(p_{dr}\left(l_{s},w\right)-{\hat{p}}_{o}\right)}^{2}}$$

In this work, we target improving the accuracy of indoor position fixes. In this case, our objective function is to minimize the positioning error, which can be obtained in both cases of multilateration and dead reckoning. For multilateration, a minimum number of BLE modules needs to be engaged in the localization procedure. Additionally, the growing error in dead reckoning has to be avoided. To achieve the above objectives, we propose a Kalman-based position fusion method, which overcomes the aforementioned limitations.

### 3.2. System Model

**Figure 1:**
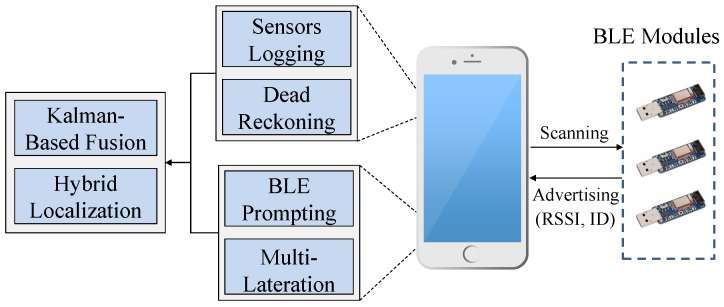
System architecture.

As explained above, several challenges stand against obtaining highly accurate indoor position fixes. This motivates us to examine a hybrid method through which the limitations of each individual method are mitigated. Figure 1 depicts the components of our proposed method. A set of BLE modules has to be fixed at specific prior-known locations. During run-time, a mobile device performs dual operations, including multilateration and dead reckoning. In the former method, the mobile device scans the wireless channel of the scattered BLE modules. If at least three BLE modules are detected, the RSSI readings are exploited to determine a coarse position fix.

Similarly, the mobile device has to activate its embedded sensors to compute the step length, heading direction and number of steps. A Kalman filter is used in this work as a fusion center to compute a fine position fix based on merging the two obtained positions. To this end, we shall first study both methods in terms of the error sources. Therefore, we investigate below several factors that negatively affect both methods, i.e., trilateration and dead reckoning.

## 4. RSSI Localization

In this section, the trilateration method in combination with RSSI measurements of BLE modules is examined. The issues of multipath and fading are described firstly. RSSI measurements are analyzed, and then, a propagation model to get the distance from the RSSI is presented. To achieve a better accuracy, different RSSI filters are applied and compared. Furthermore, the trilateration method is explained in detail, and the proposed algorithm is shown. At the end, the impact of the antenna direction and the position of the human body are respectively examined.

### 4.1. RSSI Problems

To achieve a high accuracy for localization, reliable approaches to measure RSSI are required. Therefore, it is fundamental to understand the behaviors of the measured RSSI values. Radio signal waves such as Bluetooth are analyzed in [13]. The results of this work show that the RSSI is influenced by interference from the environment, which is mainly caused by reflections, i.e., when the signal wave hits an object. These reflections can lead to multiple paths or fading signals that cause incorrect RSSI measurements.

### 4.2. RSSI Analysis

**Figure 2:**
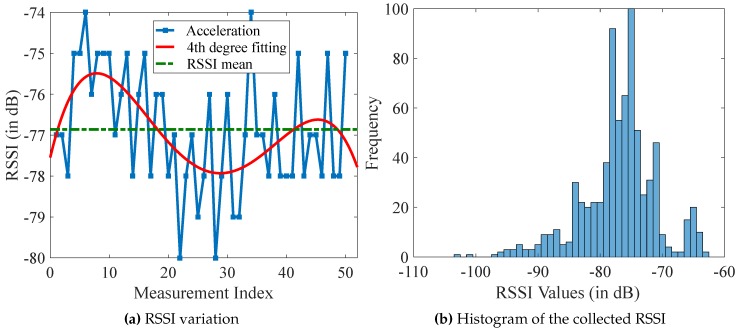
RSSI analysis for a BLE-based communication.

To analyze RSSI variations, 50 RSSI values are measured with a constant distance of one meter away from the BLE module. Figure 2a shows the expected RSSI variance within an interval of 7 dB and a standard deviation of 1.25 dB.

This experiment is repeated with seven BLE modules. The histogram in Figure 2b displays the RSSI values of all measurements. It shows that the distribution of the RSSI values spreads around −75 dB. Table 3 lists the results. The average value of all measurements is −76.72 dB, and the average standard deviation is 3.91 dB. These results show that the raw RSSI values are not reliable enough for the localization, and some filters are required to improve the stability of the RSSI values.

### 4.3. Signal Propagation Model

Before applying trilateration to calculate the position, it is necessary to estimate the distances between the mobile node and, at least, three reference nodes. The calculation is based on the relation of the distance to the RSSI. The RSSI decreases with the distance of nodes, namely the longer the distance is, the lower the RSSI becomes. Since RSSI is highly affected by multipath and fading phenomena, a model considering the environment is required. A well-known model to convert an RSSI value into distance is the log-distance path loss model [14]. This path loss model defines the path loss as the difference between the transmission power ($P_{TX}$) and the received power ($P_{RX}$).

(4) $$PL=P_{TX}-P_{RX}$$

The path loss model can also be expressed as:

(5) $$PL=PL_{d_{0}}+10n\times \log_{10}\left(\frac{d}{d_{0}}\right)+X_{\sigma }$$

where $PL_{d_{0}}$ is the RSSI reference value measured at the distance $d_{0}$. The parameter *n* is the path loss exponent, which indicates the rate of increasing path loss related to the distance. It also represents the multipath effect. Further, *d* is the actual distance to the transmitter, and $X_{\sigma }$ is a zero-mean normal random variable with standard deviation. Without fading, this variable is set to zero. The path loss model can also be expressed as [15]:

(6) $$RSSI=RSS_{d_{0}}-10n\times \log_{10}\left(\frac{d}{d_{0}}\right)+X_{\sigma }$$

where $RSS_{d_{0}}$ is the measured reference value at the distance of $d_{0}$. For further experiments in this work, $d_{0}$ is set to 1 m, so that $RSS_{d_{0}}$ represents the measured RSSI at a distance of 1 m to the transmitter. Since there is no large obstacle in the environment to be tested, shadowing is not expected, and $X_{\sigma }$ is set to zero. The path loss exponent *n* depends on the environmental conditions. Table 4 lists some typical values for *n* regarding the environment.

In Section 4.2, the reference value measured with the distance of 1 m to the transmitter is already determined as −76.72 dB. When the distance *d* is known, then *n* can be calculated by the following equation:

(7) $$n=\frac{RSS_{d_{0}}-RSS}{10\times \log_{10}\left(d\right)}$$

To get *n* from each BLE module, RSSI values are measured at different distances, i.e., 0.5 m, 1.5 m, 2 m, 2.5 m, 3 m, 3.5 m and 4 m, respectively, under the same condition. At each distance, 30 values with each BLE module are measured and averaged. Table 5 shows the average results of *n* for each BLE module.

**Figure 3:**
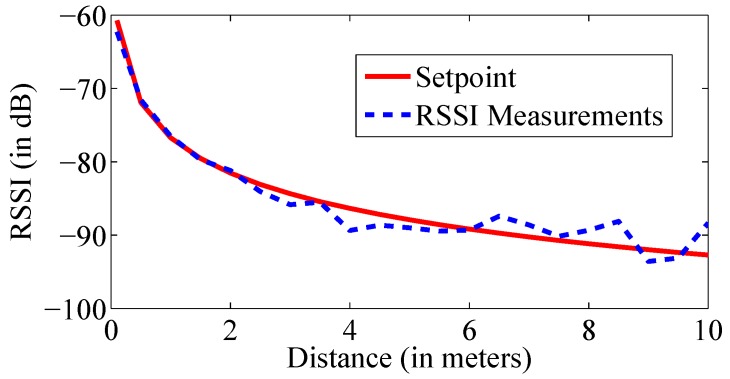
Impact of the path loss model.

The average value of n=1.63 is used for the further measurements. Figure 3 shows that the RSSI value decreases with the increasing distance. The red curve represents the theoretical curve form of the path loss model with the calculated parameters, while the blue curve shows the measured RSSI values regarding the reference distance. It is clear that the difference between both curves increases with increasing distance. Therefore, it is showed that applying the distances of less than 4 m in the RSSI-based model is more accurate.

### 4.4. RSSI Filtering

The significant variance in the RSSI measurements leads to unreliable distance calculation. To avoid this, it is necessary to filter the RSSI values. In this section, the average, the median and the Kalman filter are compared to find the best fitting filter for the RSSI measurements.

#### 4.4.1. Average and Median Filter

The only adjusting parameter for the average and the median filter is the frame size. A larger frame size might lead to a higher accuracy. However, if the frame size is too large, the measurements inside the frame becomes rapidly out-of-date. We set the frame size to 20 in this work.

#### 4.4.2. Kalman Filter

For the RSSI filtering, a one-dimensional Kalman filter is used. As previously mentioned, the Kalman filter has some parameters that have to be determined first. Looking at the RSSI values, their change is random. Therefore, the transition matrix *F* and the measurement matrix *H* are set to one. Furthermore, there is no external control input. Therefore, $B\times u_{t-1}$ is also set to zero. With these assumptions, the prediction and update phase can be shown as:

- Prediction Phase:
  (8) $${\hat{x}}_{t}^{-}={\hat{x}}_{t-1}$$
  (9) $$P_{t}^{-}=P_{t-1}+Q$$
- Update Phase:
  (10) $$K_{t}=\frac{P_{t}^{-}}{\left(P_{t}^{-}+R\right)}$$
  (11) $${\hat{x}}_{t}={\hat{x}}_{t}^{-}+K_{t}\left(z_{t}-{\hat{x}}_{t}^{-}\right)$$
  (12) $$P_{t}=\left(1-K_{t}\right)P_{t}^{-}$$

**Figure 4:**
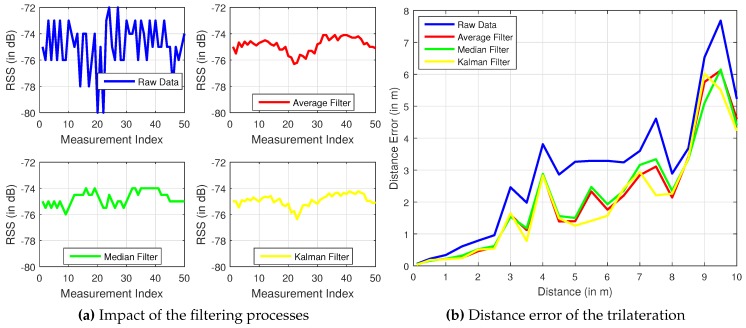
Comparative analysis of the adopted filters.

The initial values are set to ${\hat{x}}_{t}^{-}=0$ and $P_{t}^{-}=0$. The values for Q=0.065 and R=1.4 are determined experimentally. After the filters are applied, the curve form of the RSSI values is smoother than before as shown in Figure 4a. The black line shows the original RSSI measurements without any filtering. There are many jumps and variations. The average filter (green line) and the median filter (blue line) do not have such huge jumps, and their variations are not as large as the raw measurements. The Kalman filter (red line) achieves the fewest jumps and variations. The next step is to compare the three filters. RSSI values are measured at distances of every 0.5 m up to 10 m. Then, each filter is consecutively applied to the raw values. Thereafter, the distance is calculated and compared with the desired value. Correspondingly, Figure 4b shows the distance error, which was measured at different distances, of each filter.

It is shown that the distance error is increasing with an increasing measurement distance. The filtered distance error by Kalman is the lowest. As a result, the Kalman filter is applied for all following RSSI filtering in this work.

### 4.5. Trilateration Method

**Figure 5:**
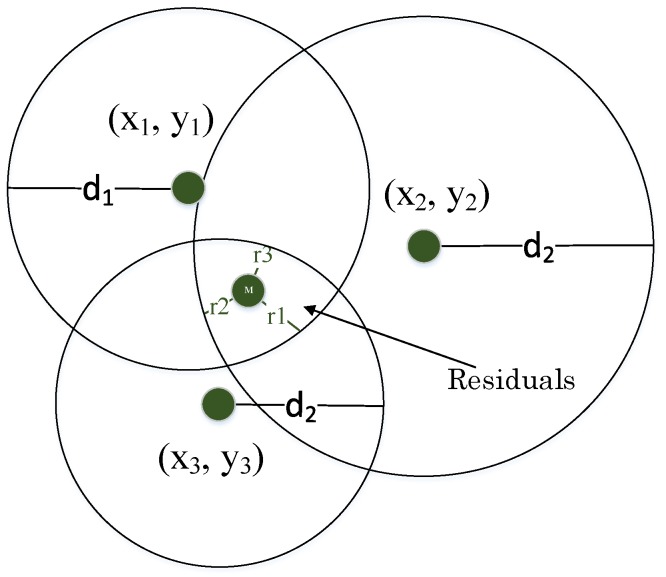
Residual values in trilateration model.

Trilateration is a traditional method to compute the unknown position of a node. At least three reference nodes with known positions are required in this method. Besides, the distances between these nodes and the mobile node are required to be known, as well. Theoretically, each reference node forms a circle around itself with the radius of the distance to the mobile node. The position of the unknown node corresponds with the intersection of these three circles. The distances in a plain to the mobile node can be expressed by the following formulas:

(13) $$d_{i}^{2}={\left(x-x_{i}\right)}^{2}+{\left(y-y_{i}\right)}^{2}$$

whereas (*x*,*y*) are the coordinates of the mobile node, which have to be computed. Further, ($x_{i}$,$y_{i}$) is the position and $d_{i}$ is the distance to the mobile node of the *i*th reference node. The equation can be solved by assuming that the three distances and position of the reference nodes are known and correct. However, the accurate distances cannot be identified due to the RSSI variations. In reality, the three circles do not intersect in one point, and there are some residuals $r_{i}$ as shown in Figure 5. The residuals are the differences between the computed $d_{i}$ and the estimated $e_{i}$ distances [16].

(14) $$r_{i}=d_{i}-e_{i}$$

Because there are three equations, but only two unknown variables, this system is over-determined and does not have one unique solution. A method to solve the problem of over-determinacy is the least square (LSQ) method. The principle of this method is to minimize the sum of the squared residuals [16]:

(15) $$\left(x,y\right)=min\left(\sum_{i=1}^{N}{\left(r_{i}\right)}^{2}\right)$$

Furthermore, in most cases, there are more than three reference nodes available so that multilateration can be applied. Using more reference nodes could increase the accuracy, since the weightiness of a measurement error produced by a single node will be reduced. Considering there are *n* reference nodes, the equation system becomes as follows:

(16) $$\begin{array}{cc} d_{1}^{2}= & {\left(x-x_{1}\right)}^{2}+{\left(y-y_{1}\right)}^{2}\\ & \vdots \\ d_{n}^{2}= & {\left(x-x_{n}\right)}^{2}+{\left(y-y_{n}\right)}^{2} \end{array}$$

By subtracting the last equation from the others one by one, the system can be linearized into:

(17) Ax=b

with:

(18) $$\begin{array}{c} A=\left[\begin{array}{c} 2\left(x_{1}-x_{n}\right)\;2\left(y_{1}-y_{n}\right)\\ \vdots \;\;\vdots \\ 2\left(x_{n-1}-x_{n}\right)\;2\left(y_{n-1}-y_{n}\right) \end{array}\right] \end{array}$$

(19) $$\begin{array}{c} x=\left[\begin{array}{c} \hat{x}\\ \hat{y} \end{array}\right] \end{array}$$

(20) $$\begin{array}{c} b=\left[\begin{array}{c} x_{1}^{2}-x_{n}^{2}+y_{1}^{2}-y_{n}^{2}+d_{n}^{2}-d_{1}^{2}\\ \vdots \\ x_{n-1}^{2}-x_{n}^{2}+y_{n-1}^{2}-y_{n}^{2}+d_{n-1}^{2}-d_{1}^{2} \end{array}\right] \end{array}$$

This constraint system can be solved by the mentioned LSQ method:

(21) $$x={\left(A^{T}A\right)}^{-1}\left(A^{T}b\right)$$

### 4.6. Proposed Algorithm

In this section, we develop a smartphone-based application for indoor localization. To improve the accuracy of the position, we implement a hybrid localization scheme with Kalman-based fusion, despite the aforementioned methods. Algorithm 1 describes the general procedure of the developed application using the trilateration method. As soon as a new RSSI is measured, a new thread starts. The measured RSSI is required to be checked if it belongs to one of the eight registered BLE modules. If the RSSI is accepted, the Kalman filter is applied. Then, the distance is calculated and saved. Since the position shall only be calculated with actual distances, the algorithm searches for saved distances older than one second and deletes them. At least three distances are required to apply the trilateration method. Therefore, the algorithm loops in the procedure of reading RSSI until the threshold $\tau_{1}=3$ is reached.
**Algorithm 1** BLE-driven trilateration method.1: **while** accepted distances i≥ Threshold $\tau_{1}$
**do**2:  **collect** the RSSI readings from *n* BLE modules3:  **if** the RSSI is **registered then**4.   **apply** the Kalman filter5:   **compute** the distance $d_{i}$6:  **end if**7:  **if**
$d_{i}>$ Threshold $\tau_{2}$
**then ignore**
$d_{i}$8:   **go to** Step 29:  **end if**10: **end while**11: **apply** the multilateration for *i* distances12: **apply** the average filter and **update** the position

As explained in Section 4.3, considering the impact of the path loss model, applying distances less than 4 m in the RSSI-based model is more accurate. Therefore, the algorithm searches for all saved distances less than or equal to 4 m and applies the trilateration or multilateration methods if three or more distances are found. Distances, longer than thresholds ($\tau_{2}=4$ m), are ignored. If not at least three distances are found, then the range of accepted distances ($\tau_{2}$) is increased by one meter, and the search for three accepted distances is repeated. If the range of accepted distances is larger than 10 m and not enough distances are available, then the thread stops, and no new position is provided. If the trilateration method is applied successfully, then the average filter is applied, and a new position is provided.

### 4.7. RSSI Impacting Factors

There are different reasons for RSSI variation. In this section, the impacts of the antenna direction of mobile devices and the human body on the RSSI variation are analyzed. For the antenna direction, RSSI is measured twice from three different positions. During the first experiment, the antenna of the mobile device is oriented towards the BLE module. During the second experiment, the antenna is oriented away from the BLE module. Table 6 shows the results of the measurements. These measurements are repeated with two further BLE modules.

The results clearly show that there is a difference of 1.5 dB–2.5 dB if the antenna of the mobile node is not oriented directly toward the transmitter. This phenomenon is one problem that reduces the accuracy and has to be considered in the future.

Another phenomenon influencing the RSSI values is the blocking of obstacles. Depending on the material of the obstacle, the extent of influence varies. One non-negligible material absorbing the signal strength is water [17]. Since the human body contains about 50%–60% water [18], it can also be seen as an obstacle preventing the line-of-sight (LoS). The RSSI values are measured twice from different distances. The antenna of the receiver is always oriented towards the BLE module so that the antenna direction does not influence the measurements. At the first run, the user is standing behind the mobile device, so that there is an LoS between transmitter and receiver. At the second run, the user is standing between the mobile device and the BLE module, so that there is no-line-of-sight (NLoS). Table 7 shows the results of these measurements.

At small distances, e.g., one meter, the difference between the RSSI measurement is very small. However, with growing distance, the difference between the measurements with LoS and NLoS is increasing. In general, the calculated RSSI with LoS is smaller than with NLoS. This experiment shows that the human body blocks the LoS especially with larger distances between transmitter and receiver.

## 5. Dead Reckoning

In this section, we investigate the dead reckoning method together with the internal sensors of a mobile device. First, we perform a noise analysis and explain the possibility of detecting steps in detail. Then, we present different methods to determine the step length. In the end, the impact of the heading direction is also analyzed. For the following experiments, a tablet, i.e., Samsung Galaxy TAB 3 10.1 P5210, is utilized.

### 5.1. Sensors Noise Analysis

Since all of the following estimations are based on sensor measurements, it is highly required that the sensors work precisely. Therefore, a noise analysis for each sensor is carried out. During the tests, the tablet is attached stationary on a table. From each sensor, 500 measurements are used for the analysis.

#### 5.1.1. Accelerometer Sensor

**Figure 6:**
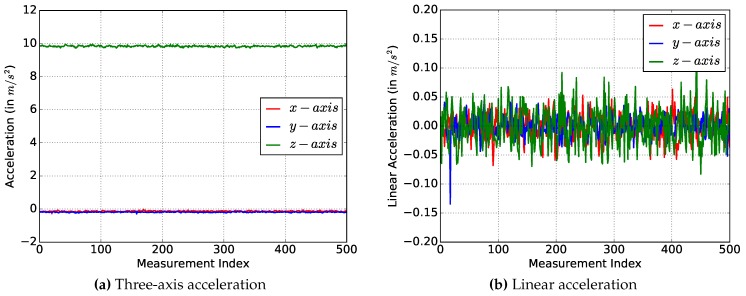
Acceleration analysis.

Most smartphones are equipped with an internal three-axis accelerometer, which can measure the acceleration force along the x-, y- and z-axis. If the device is placed stationary on a table, then the expected measurements for the x- and y-axis are 0 m/s${}^{2}$, since no acceleration is applied along these axes. On the z-axis, Earth gravity applies, so that a value of 9.81 m/s${}^{2}$ is expected. Figure 6a shows the results of the three axes.

As expected, the average values of the x- and y-axis are nearly 0 m/s${}^{2}$ and of the z-axis are about 9.8 m/s${}^{2}$. The average values of the measurements and the standard deviations are displayed in Table 8. Since the standard deviation of each axis is very low, we believe that the measurements are very precise.

The linear acceleration is a measured acceleration with removed gravity acceleration. The gravity acceleration of 9.8 m/s${}^{2}$ can be isolated by applying a low-pass filter. By applying an additional high-pass filter, the gravity acceleration can then be subtracted from the measured acceleration. The following equations for the low-pass and high-pass filters are provided by Android [19] to convert the acceleration into linear accelerations.

(22) gravity=α×gravity+(1−α)×acc;

(23) accLin=acc−gravity;

where α is a smoothing constant, which is advised to be 0.8 by Android. First of all, the gravity accelerations to each axis are calculated, and then, they are subtracted from the measured acceleration. Figure 6b shows the time curve of the linear accelerations. Compared to the measured acceleration, the values of the z-axis is now around 0 m/s${}^{2}$ (Table 9).

#### 5.1.2. Magnetometer Measurements

**Figure 7:**
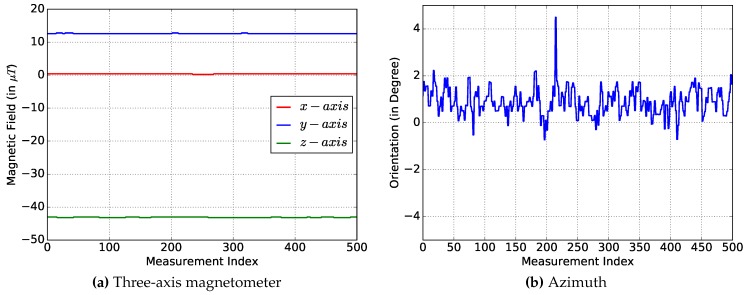
Acceleration analysis.

The three-axis magnetometer is used to measure the magnetic field of the Earth. The expected magnitude of the Earth’s magnetic field in Europe is about 48μT [20]. Figure 7a shows the measurements of the three axes. The results including the magnitude are presented in Table 10. The magnitude *M* is calculated as the absolute value of each axis:

$$M=\sqrt{x^{2}+y^{2}+z^{2}}=44.9\;\mu T$$

The results show a magnitude of the Earth’s magnetic field of about 45μT and a standard deviation of about 0.1μT. The results are reasonable considering the natural fluctuation in the magnetic field and the influence of the environment.

#### 5.1.3. Orientation

The orientation sensor is a virtual sensor provided by Android to output the smartphone’s heading direction. The direction is computed by the fusion values of the accelerometer and the magnetometer. The outputs are the rotation around the x- (pitch), y- (roll) and z- (azimuth) axis. For the dead reckoning method, only the azimuth is required [21]. The measurement of the azimuth is depicted in Figure 7b. The standard deviation in Table 11 of these measurements is higher compared with the accelerometer measurements. However, even an orientation error of 6° could lead to acceptable position estimations (see Section 5.5).

### 5.2. Gait Characteristics

For the step detection, it is necessary to explore the characteristics of a gait. Gait analysis in [22] shows that the basic human walk is periodical and noticeable. During normal walking speed, the center of gravity of the body changes smoothly in the horizontal, as well as in vertical direction. Each step can be divided into a stance phase and a swing phase. During the stance phase that constitutes about 62% of the entire gait, the feet are in contact with the ground. The swing phase starts with the foot leaving the ground and ends when the foot touches the ground again. During each gait cycle, the center of gravity performs two oscillations, which creates two high and two low peaks. The high peak occurs at the mid-stance and the mid-swing [23].

### 5.3. Step Counter

**Figure 8:**
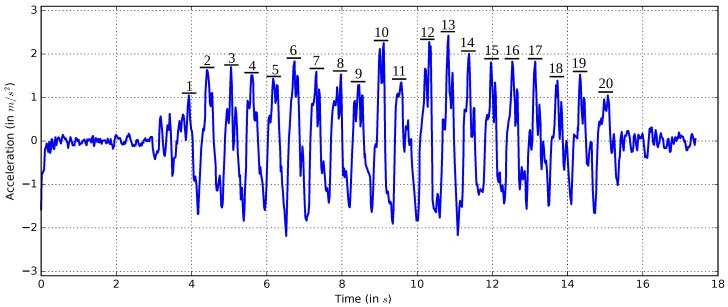
Vertical acceleration record of 20 steps.

On the basis of the gait characteristics awareness, it is possible to detect steps by observing the vertical acceleration. A step is defined as a movement made by lifting one foot and putting it down a step length away. First, measurements of the vertical acceleration are performed. Figure 8 shows the wave form of the vertical acceleration recorded during 20 steps.

There are different methods to identify a step. One of the simplest ways is to identify a step through a high peak. However, because the measurements result in multiple peaks and the amplitude varies, this method is not reliable enough. An advanced method [24] uses the fact that a high peak is always followed by a low peak. A high and low threshold are set to recognize the peaks, and a step is counted when a high peak is followed by a low peak. However, unintended movements of the smartphone can lead to incorrect step detection. Further observations [25] show that a step is also characterized by its duration time. The typical time between the high and low peak takes about 150 ms–400 ms. This assumption is based on the fact that a human normally takes two steps per second in normal walking speed.

Similar to [25], a step is detected when three conditions are all met. Firstly, the accelerometer measurements have to exceed the upper threshold. Secondly, the accelerometer measurements have to fall below the lower threshold. Thirdly, the time between the exceedance of the upper threshold and the fall below the lower threshold must be between 150 ms and 400 ms.

In Figure 8, it can be observed that the threshold of the high peak must be at least 1 m/s${}^{2}$ or smaller, and the low threshold must be −1 m/s${}^{2}$ or higher. To find the best thresholds, some step measurements are carried out. In each test, the user walks 50 steps. The results in Table 12 show that a threshold of 0.7 obtains the lowest error rate.

As previously mentioned, the Android OS provides a step detector. Although the step detector works with Android 4.4, it also needs special motion-tracking hardware. However, only a handful of devices such as Nexus 5 or Moto X have such hardware. Therefore, the Android step detector is not examined any further.

### 5.4. Step Length Estimation

For predicting the next position, it is necessary to know the step length. The step length depends on many factors like walking speed, walking style and personal height. Therefore, there are some different solutions for adjusting the step length. One solution is to set the step length to the average value of the human step length. The average value of step length of men is 0.78 m, and that of women is 0.70 m. The average value of both values is 0.74 m [26].

Another way of step length estimation is that it could be individually calculated by the height of a human being. Therefore, at the first use of the indoor localization system, the height must be given. According to [26], the step length is calculated by multiplying the height by a factor resulting in a man’s step length.

Nevertheless, the step length is not constant and depends, for example, on the walking speed and the individual walking style. Typically, the step length increases with increasing walking speed. Another solution [27] updates the steps length during the walk. The step length is calculated by using walking speed, walking frequency and acceleration. The speed is calculated by the integral of the acceleration. However, calculating the speed from the accelerometer measurements is very error-prone. Comparing the presented solutions, the online estimation seems to be not very reliable. Furthermore, the approach of entering the height is not very user-friendly since it requires explicit action from the user. The first solution, setting the step length to the average value of 0.74 m, is the most practical solution from this perspective. In the following, it is assumed that the user walks slowly with an average speed of about 1 m/s so that the step length is quite constant. For these reasons, the approach of setting the step length to the fixed value of 0.74 m is used.

### 5.5. Heading Estimation

Since users are not always walking strictly straightforward, the heading of the smartphone must also be known. To get the heading direction, the Android orientation sensor is applied. An offset is added to the value so that the heading along the positive x-axis of the testbed coordinate system is $0^{\circ }$/$360^{\circ }$. To determine the accuracy of the heading, eight different tests are carried out. Each test comprises 1000 single measurements. The results of these tests are shown in Table 13.

The results show that the average error is no larger than $6^{\circ }$. To see the effect of this error, the following exposition is done: When the user takes a step along the x-axis, it is expected that the x-value is increased by 0.74 m, and the y-value does not change. If the heading measurement is measured incorrectly by $6^{\circ }$, the following equation shows the distance error ϵ.

(24) $$x_{t}=x_{t-1}+0.74\;m\times \cos\left(6^{\circ }\right)=0.736\;m$$

(25) $$y_{t}=y_{t-1}+0.74\;m\times \sin\left(6^{\circ }\right)=0.077\;m$$

(26) $$\epsilon =\sqrt{{\left(0.74\;m-0.736\;m\right)}^{2}+{\left(0-0.077\;m\right)}^{2}}=0.08\;m$$

The calculation shows that even a deviation of 6${}^{\circ }$ leads to only a distance error of 8 cm, which is acceptable for the further calculations. Nevertheless, a disadvantage is the vulnerability of the magnetometer to environment influence over time. One solution to this problem could be calibrating the magnetometer regularly.

## 6. Hybrid Indoor Localization

In this section, we elaborate on the position fusion mechanism using the Kalman filter. The positions of mobile devices are deduced from a combination of the trilateration and the dead reckoning methods. The motivation behind our proposed hybrid positioning method centers on exploiting the advantages of each method and simultaneously dodging possible disadvantages. For instance, the position estimation using the trilateration method seems to be not as accurate as dead reckoning. However, the average error due to trilateration remains constant. On the other hand, dead reckoning produces highly accurate position fixes at the beginning of the measurements. Nevertheless, the dead reckoning performance continuously drops due to accumulative sensing errors.

**Figure 9:**
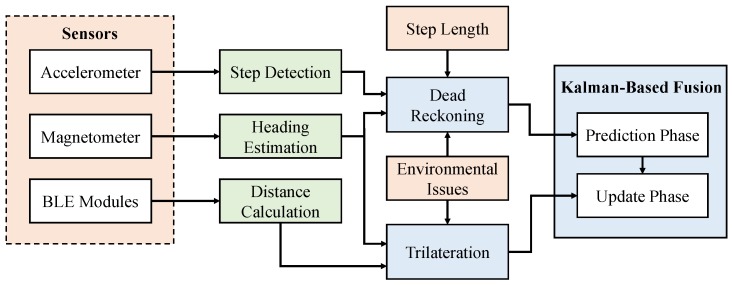
Architecture of the proposed Kalman-based fusion method.

Figure 9 depicts the processing pipeline of our proposed method. As previously mentioned, dead reckoning generates a position fix provided that the number of steps and the user orientation are know. Similarly, trilateration requires several distance measures between the mobile device and the surrounding BLE modules. Both dead reckoning and trilateration have to be rectified from deviations due to environmental issues such as multipath fading. Below, we discuss the integration of heading with the trilateration method before we describe the fusion method in more detail.

### 6.1. Heading-Enhanced Trilateration

The LoS is a crucial condition for accurate localization. With only the BLE modules, it is not trivial to detect the heading of the mobile device. When integrating the trilateration method with internal sensors of the mobile device, the heading direction can be determined and taken into account while estimating the position fix. Two different methods are examined to find the best solution for this scenario, including (1) adding offsets and (2) adapting the propagation model. The former solution considers adding or subtracting an offset to the position depending on the heading direction. The first experiments showed that the measured RSSI values with the NLoS setting are lower than the typical case. Consequently, the calculated distance becomes larger than the real distance. In other words, the estimated distances from devices “behind the user” are larger than the distances in reality. Thus, the estimated position is moved some centimeters before the user. Through extensive experiments, we found a position shift along the y-axis by approximately 30 cm. Therefore, the offset is set to ±30 cm depending on the heading direction. Since the corridor, in our scenario, is not wide relative to its length, the offset is solely added to the y-axis.

The core idea behind the second method is to adapt the propagation model according the user orientation. As a matter of fact, distance measures typically depend on both the actual position and the heading direction. At the outset, we individually determine the model parameters $RSS_{d_{0}}$ and *n* for each static BLE module. This calculations should be performed twice in the case of LoS and NLoS conditions. Subsequently, all distances to BLE modules behind the user are calculated with the NLoS-parameters; whereas, the distances to BLE modules, which are in front of the user, are calculated with the LoS parameters. Table 14 and Table 15 summarize the calculated model parameters $RSS_{d_{0}}$ and *n* values for both cases. The differences between the calculated parameters $RSS_{d_{0}}$ and *n* mainly vary with the environment characteristics.

### 6.2. Kalman-Based Fusion

A Kalman filter is chosen to integrate trilateration and dead reckoning, thanks to the efficiency and simplicity of the algorithm. Since the position normally consists of two variables, a two-dimensional Kalman filter is applied. It is assumed that the state vector ${\hat{x}}_{t}=\left[\begin{array}{c} x\\ y \end{array}\right]$ represents the 2D coordinates of the user, i.e., his/her position. The state equation can then be expressed as:

(27) $${\hat{x}}_{t}=\left(F\times {\hat{x}}_{t-1}\right)+\left(B\times u_{t}\right)$$

where *F* and *B* are identity matrices and $u_{t}=L\times \left[\begin{array}{c} \sin(\theta )\\ \cos(\theta ) \end{array}\right]$ is the input vector derived from the dead reckoning method. In this case, the term *L* is the step length, and θ is the heading direction. The measurement model is obtained from the trilateration-based position. The trilateration-based position $\left[\begin{array}{c} x_{T}\\ y_{T} \end{array}\right]$ is more often estimated than the dead reckoning-based position. Therefore, we determine an average value of *N* trilateration-based positions since the last Kalman update. The measurement model can be expressed as,

(28) $${\hat{z}}_{t}=\frac{1}{N}\sum_{i=1}^{N}\left[\begin{array}{c} x_{T}\left(i\right)\\ y_{T}\left(i\right) \end{array}\right]$$

Since the position have to be regularly updated, the Kalman filter updates the position whenever a new step is detected or periodically with a period length of one second. The Kalman filter has a prediction and an update phase. For the prediction of the new position, the dead reckoning method is used. During the update phase, the Kalman filter merges the predicted position with the average value of the trilateration-based positions. In the prediction phase, a position can be predicted as expressed by Equations (29) and (30).

(29) $${\hat{x}}_{t}={\hat{x}}_{t-1}+u_{t}$$

(30) $$P_{t}^{-}=P_{t-1}+Q$$

where *Q* is the covariance of the noise from the dead reckoning method. In the update phase, both positions are merged. Accordingly, the fused position $P_{t}$ is expressed in terms of the Kalman gain *K*, the identity matrices *H* and *I* and the covariance of the noise from the trilateration method *R*, as denoted in Equations (31) and (33).

(31) $$K_{t}=P_{t}^{-}\times H^{T}{\left(HP_{t}^{-}H^{T}+R\right)}^{{-}^{1}}$$

(32) $${\hat{x}}_{t}={\hat{x}}_{t}^{-}+K_{t}\left(z_{t}-H{\hat{x}}_{t}^{-}\right)$$

(33) $$P_{t}=\left(I-K_{t}H\right)P_{t}^{-}$$

The Kalman gain *K* determines the contribution of each method to the fused position. The main parameter that highly affects the Kalman gain *K* is the covariance of the noise from the trilateration method. Different values for *R* and *Q* are experimentally tested. According to our experiments, the highest positioning accuracy is obtained by using R=1.25 and Q=0.005. Below, we elaborate on the designed testbed before we describe the results of our experiments to evaluate the fusion method. Moreover, we provide a comparative study between the proposed fusion method and the two baseline methods including trilateration and dead reckoning.

## 7. Performance Evaluation

In this section, the performance of the proposed Kalman-based fusion system is evaluated. First, we describe a testbed composed of a set of BLE modules and a mobile device. Afterwards, the performance of both the trilateration and the dead reckoning is evaluated at static and dynamic positions. Additionally, the influence of the knowledge of the environmental context is examined. Finally, the accuracy of the fusion method is compared to the trilateration and the dead reckoning methods.

### 7.1. Experiment Setup

**Figure 10:**
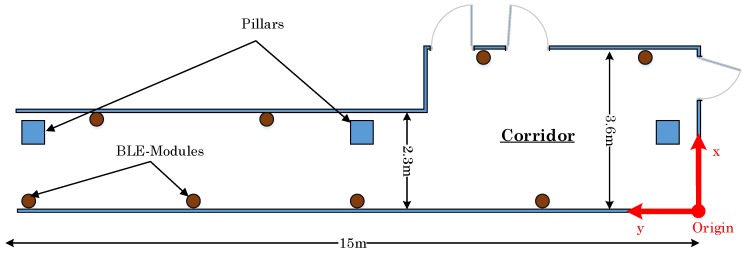
Layout of the implemented testbed.

Figure 10 depicts the testbed engineered for the positioning performance. The brown points represent eight BLE modules, which are placed in the corridor. The decision of these BLE modules placement is made such that the corridor is entirely covered by BLE signals. The test area of the corridor has a length of 15 m. The first 6 m (from the right) of the corridor has a width of 3.6 m. After 6 m, the hallway narrows to 2.3 m. The point in the lower right corner in the figure is chosen as the coordinate system origin.

The BLE modules are placed at a height of 2.65 m. The main reason for the high position is the reduced disturbance through people walking by. Since a 2D trilateration method is applied, the 3D model of the room has to be broken down to a 2D-model. Hence, the distance $d_{r}$ from the mobile device to the position at the wall below the BLE module is required. The distance $d_{r}$ is determined in terms of the measured distance $d_{m}$ and the difference $h_{\Delta }$ between the BLE module’s and the mobile device’s height, as given by:

(34) $$d_{r}=\sqrt{d_{m}^{2}-h_{\Delta }^{2}}.$$

Since the testbed is fixed, the position value is also limited through the environment context. It is assumed that users are not walking closer than 0.5 m to the wall. Therefore, it is not possible that people can walk through walls or outside the testbed. In our experiments, the BLE modules Bluefruit LE Friend (BLE 4.0) are adopted. These BLE modules use the Nordic nRF51822 chipset. Further, the developed application for all position estimations is running on a Samsung Galaxy TAB 10.1. This application measures the RSSI and saves the values for the evaluation in text files.

### 7.2. Step Detection

The performance of dead reckoning mainly relies on the step detection. Hence, the step detector application is foremost evaluated. To test the accuracy of the step detection, four volunteers have walked 50 steps along a straight path while counting the steps. After the walk, the detected steps are compared with the expected 50 steps. To have more reliable measurements, each volunteer has performed this test twice using two mobile devices, including the Motorola Moto G and the tablet Samsung Galaxy TAB 10.1. The results of the step detection test are listed in Table 16 and Table 17.

As can be seen in the tables, the step detector using the Samsung tablet is more accurate relative to the smartphone. Furthermore, the smartphone tends to count easily false steps when the user moves or turns the smartphone. One reason why this happens may depend on the bearing of the mobile device. Generally, users carry their smartphone in one hand, while the tablet is carried in both hands. Hence, the tablet is relatively more stable and is not accelerated that much in the vertical direction. As a result, we need to set a larger threshold to avoid false step detection when using a smartphone. Accordingly, we repeat the smartphone-based experiment after increasing the threshold from 0.75 to 0.9, as depicted in Table 18.

As shown in Table 18, the step detection is improved when increasing the threshold. To sum up, both step counter tests achieve an average accuracy of 96.68%. Nevertheless, both devices have false positives when a user is swiftly changing the heading direction. Additionally, false positives occur when mobile users walks too fast, while false negatives occur when users walks too slow.

### 7.3. Position Fusion

**Figure 11:**
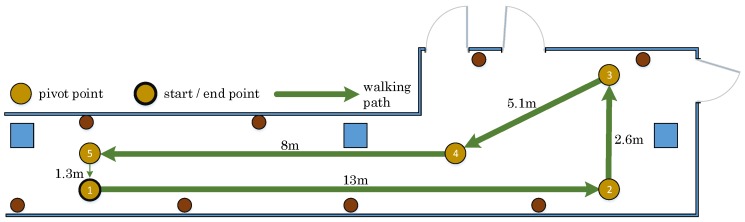
Walking path during the experiments.

In this section, we evaluate the fusion method compared to the baseline methods, including trilateration and dead reckoning. Specifically, dead reckoning requires an initial value of the start position. In each test, a volunteer walks along a determined path with a length of about 30 m, as shown in Figure 11. During the experiment, the position is estimated using three different methods. The experiments are conducted twice using two settings, with and without considering the context information. Below, we discuss these two sets of experiments.

#### 7.3.1. Performance without Context Information

**Figure 12:**
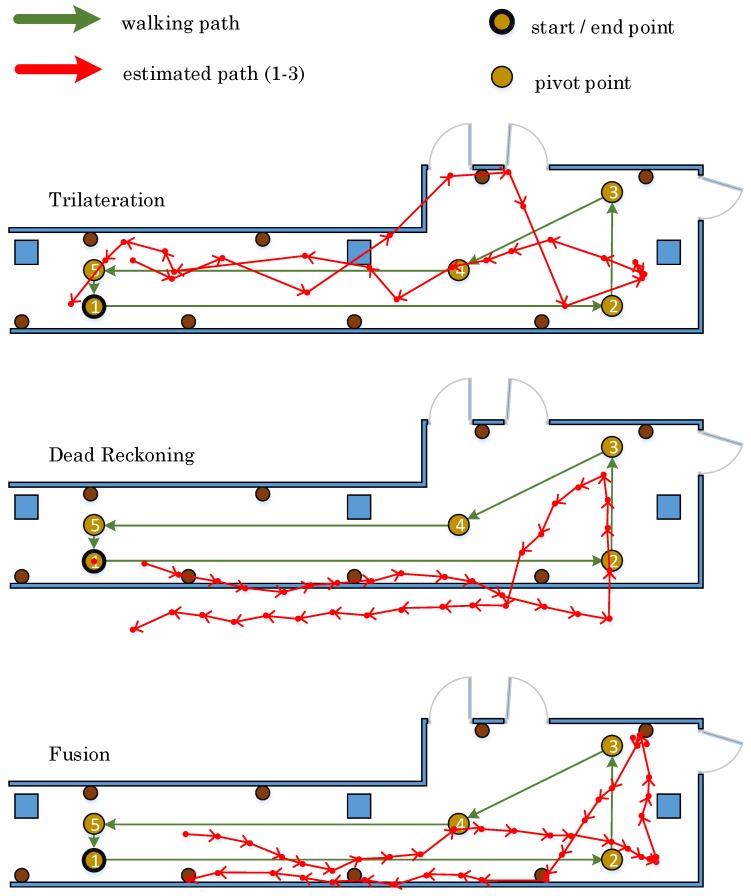
The detected walking paths without context information.

In this set of experiments, the position estimation does not consider the environment context. Hence, it might be possible that the position of a user is displayed outside of the corridor. Figure 12 shows the measurements exemplary of one walked path for each method. It is obvious that the estimated path of the trilateration method differs from the actual walked path. The estimated path with the dead reckoning method conforms rather to the walked path, but it is shifted outside of the corridor. The estimated path using the proposed fusion method mostly matches with the walked path. These measurements are repeated three times to increase our confidence in the obtained results. Besides, we are also interested in the position accuracy of the final stop position after each single walk. Table 19 demonstrates the accuracies of the estimated final position after each trial using three different methods, respectively. The average accuracy addresses that the trilateration method is the best one out of three in terms of estimating the final position, with only an average difference of 0.73 m, even though the estimated path of the trilateration method differs the most from the real walked path, as shown in Figure 12.

#### 7.3.2. Context Information-Enhanced Positioning

**Figure 13:**
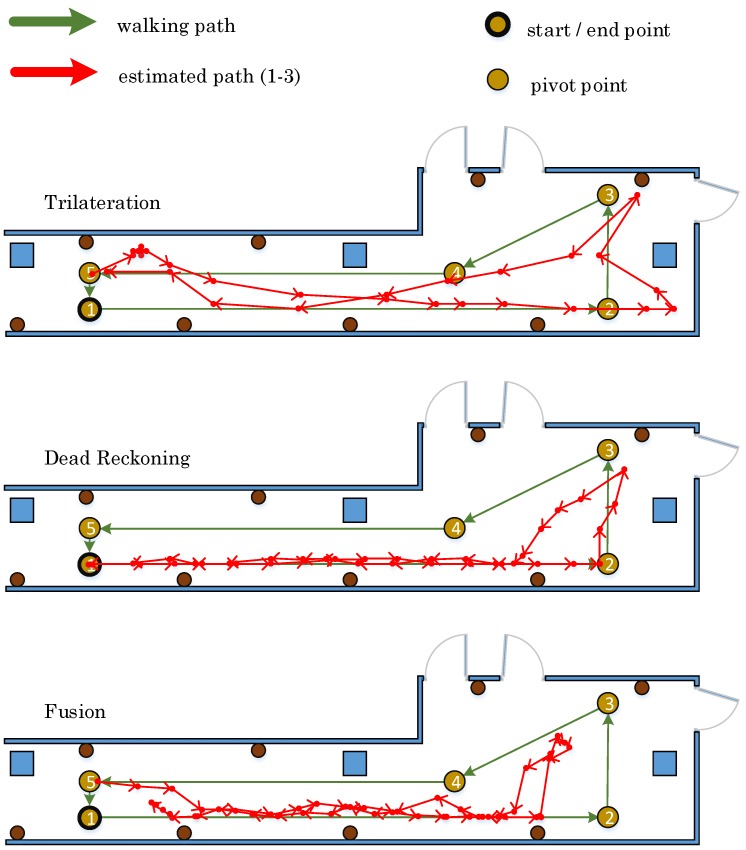
The detected walking paths with context information.

The resultant low accuracy of the dead reckoning method emerges since the positioning error is growing over time. To limit this error, the estimated positions are rectified using a number of constraints extracted from the environment context information. At the outset, the path is walked once. The pathways of the estimated paths, exemplary for one walk for each method, are displayed in Figure 13. Through limiting the position in a bounding box representing the corridor layout, all three estimated paths precisely match the real walked path. Specifically, the path of dead reckoning gains an improved trajectory when adopting the constraints. Similarly, as depicted in Table 20, the accuracy of estimating the final position is highly improved by using the dead reckoning with the environmental context; whereas, the accuracy of the trilateration method and the fusion method does not vary much.

### 7.4. Results

The evaluations show that the three methods investigated in this article have their advantages and disadvantages. Dead reckoning has a positioning accuracy of about 1 m when considering the context information. Additionally, the trajectory estimated by dead reckoning is very close to the real trajectory. Another advantage of dead reckoning lies in its ability to produce position fixes without additional hardware. However, an initial start position has to be determined at the beginning, which is not trivial to be supported in many cases. Furthermore, the error due to dead reckoning grows over time especially in large spaces. Furthermore, the slightly occurring false positives or false negatives during step detection affect the obtained positioning accuracy. Another severe problem is the interference of the magnetometer. During our evaluations, we calibrate the magnetometer before each use to mitigate this interference.

The trilateration method, on the other hand, overcomes the problem of the growing error and the requirement of a start position. Trilateration shows an almost low error below 1 m. Nevertheless, the trilateration method suffers from other problems. Although, the error is mainly constant, the position estimation is relatively jumpy. For the RSSI measurements, additional BLE modules are required. Further, the Android APP consumes much energy for scanning the BLE modules. Because users’ orientation has to be performed in each position fix, the trilateration method also suffers from the magnetometer interference.

The Kalman-based fusion method overcomes some of the aforementioned problems. The average accuracy of 1 m stays almost constant. Additionally, no start position is required, and the position estimation is not as jumpy as by using the trilateration method. However, the fusion method also suffers from interference of the magnetometer, and it requires additional BLE modules. Furthermore, the energy required to generate a position fix is higher than the baseline methods due to combining two position fixes. Table 21 summarizes the advantages and disadvantages of each method.

## 8. Conclusions

In this paper, we proposed a novel positioning method based on the concept of data fusion. Kalman filtering is used to determine position fixes emerging from combining trilateration-based fixes and dead reckoning-based fixes. A number of experiments is performed to examine the positioning accuracy and the limitations of each method. The results concluded that position fusion generates highly stable position fixes with an accuracy of less than one meter. Considering the context information, such as the corridor width and length, significantly improves the obtained results. As an outlook, we plan to extend the experiments via evaluating other properties such as energy consumption and the scalability. Additionally, we plan to provide a comparative study of the impacts of the Kalman-based fusion method with other methods, such as Bayesian inference [28].

## Figures and Tables

**Table 1:** Comparison of different wireless communication technologies

Characteristic	WiFi	Classic Bluetooth	BLE
Signal Rate	54 Mbps	1 Mbps	720 Kbps
Normal Range	100 m	10 m	10 m
Transmission Power	20 dBm	10 dBm	1 dBm
Energy Consumption	100–50 mA	57 mA	15 mA
Hardware Cost	high	medium	low

**Table 2:** Accuracy of different approaches [12].

Solution	Accuracy	Advantages/Disadvantages
Active Badge	room size	− low accuracy
		− additional hardware
Cricket	10 cm	+ good accuracy
		− high hardware cost
		− additional hardware
RADAR	2–3 m	+ low hardware cost
		− varying RSSI
		− database creation
Smartphone + Internal Sensors	2–3 m	+ low hardware cost
		− emerging error
		− sensor interference
Smartphone + WiFi RSSI-Fingerprint	1–2 m	+ good accuracy
		+ low hardware cost
		− varying RSSI
		− database creation
Smartphone + Internal Sensors + WiFi RSSI-Trilateration	1.5 m	+ good accuracy
		+ low hardware cost
		+ constant error
		− varying RSSI
		− sensor interference

**Table 3:** Average RSSI values.

BLE Module No.	1	2	3	4	5	6	7	8	Average
Average (in dB)	−74.08	−75.36	−71.00	−80.73	−77.65	−81.25	−79.63	−74.06	−76.72
Standard Deviation	2.78	2.23	5.84	5.71	2.97	5.71	3.43	2.57	3.91

**Table 4:** Path loss exponents for different environments.

Environment	Path Loss Exponent *n*
Free Space	2
Urban Area Cellular Radio	2.7–3.5
Shadowed Urban Cellular Radio	3–5
Line-of-Sight in Building	1.6–1.8
Obstruction in Building	4–6
Obstruction in Factories	2–3

**Table 5:** Calculations of the loss path exponent *n*.

BLE Module	1	2	3	4	5	6	7	8	Average
*n*	2.72	1.60	2.42	0.75	1.49	0.77	1.61	1.71	1.63

**Table 6:** RSSI measurements depending on the antenna direction.

Distance	1 m	2.5 m	4 m
Oriented towards Receiver	−80.54 dB	−79.74 dB	−86.86 dB
Not Oriented towards Receiver	−82.16 dB	−82.95 dB	−88.59 dB
Difference	1.62 dB	2.41 dB	1.73 dB

**Table 7:** RSSI values with LoS and no-line-of-sight (NLoS) scenarios.

Distance	1 m	2.5 m	4 m
LoS	−79.96 dB	−76.46 dB	−81.40 dB
NLoS	−81.65 dB	−79.59 dB	−87.32 dB
Difference	1.69 dB	3.13 dB	5.92 dB

**Table 8:** Acceleration measurements.

	x-Axis	y-Axis	z-Axis
Average in m/s${}^{2}$	−0.1293	−0.1895	9.8397
Standard Deviation in m/s${}^{2}$	0.0202	0.0156	0.03244

**Table 9:** Linear accelerometer measurements.

	x-Axis	y-Axis	z-Axis
Average in m/s${}^{2}$	−0.0003	0.0003	0.0001
Standard Deviation in m/s${}^{2}$	0.0171	0.0131	0.0264

**Table 10:** Magnetometer measurements.

	x-Axis	y-Axis	z-Axis	Magnitude
Average in μT	0.3864	12.6172	−43.1112	44.9213
Standard Deviation in μT	0.0254	0.0314	0.0987	0.0966

**Table 11:** Azimuth measurements.

	Azimuth
Average in ${}^{\circ }$	0.8936
Standard Deviation in ${}^{\circ }$	0.4581

**Table 12:** Threshold examination.

Threshold	Run 1	Run 2	Run 3	Run 4	Average Step Error
±0.65	52	52	49	51	1.5
±0.7	48	49	50	51	1
±0.75	53	49	47	48	2.2
±0.8	47	51	35	47	5.5

**Table 13:** Heading measurement values.

Desired Heading	0°/360°	45°	90°	135°	180°	225°	270°	315°
Average	$359.56^{\circ }$	$47.84^{\circ }$	$93.31^{\circ }$	$134.02^{\circ }$	$185.36^{\circ }$	$225.01^{\circ }$	$271.81^{\circ }$	$313.84^{\circ }$
Standard Deviation	$0.81^{\circ }$	$2.85^{\circ }$	$3.11^{\circ }$	$1.56^{\circ }$	$5.36^{\circ }$	$0.77^{\circ }$	$1.83^{\circ }$	$1.28^{\circ }$

**Table 14:** The propagation model parameters in the case of the LoS condition.

BLE Module No.	1	2	3	4	5	6	7	8
$RSS_{d_{0}}$	−74.62	−65.16	−71.3	−78.6	−78	−80.46	−76.74	−72.25
*n*	2.57	1.42	2.47	1.07	1.04	0.05	1.94	1.68

**Table 15:** The propagation model parameters in the case of the NLoS condition.

BLE Module No.	1	2	3	4	5	6	7	8
$RSS_{d_{0}}$	−76.86	−76.1	−76.84	−82.86	−77.3	−82.04	−82.52	−75.67
*n*	2.85	1.78	2.36	0.42	1.93	1.48	1.26	1.74

**Table 16:** Step detection using a Motorola smartphone.

Volunteer No.	Walk 1	Walk 2	Walk 3	Average Error in Steps	Accuracy
1	55	59	61	8.3	83.3%
2	52	61	58	3.7	92.7%
3	50	51	52	1.0	98.0%
4	51	51	51	1.0	98.0%
Average				4.3	91.4%

**Table 17:** Step detection using a Samsung tablet.

Volunteer No.	Walk 1	Walk 2	Walk 3	Average Error in Steps	Accuracy
1	51	52	53	2.0	96.0%
2	49	51	50	0.7	98.7%
3	50	56	53	3.0	94.0%
4	50	50	53	1.0	98.0%
Average				1.7	96.7%

**Table 18:** Step detection using a Motorola smartphone with higher threshold.

Volunteer No.	Walk 1	Walk 2	Walk 3	Average Error in Steps	Accuracy
1	55	46	46	4.3	91.3%
2	50	49	50	0.3	99.3%
3	50	51	51	0.7	98.7%
4	49	53	50	1.3	97.3%
Average				1.7	96.7%

**Table 19:** Positioning accuracy of final position after each trial without considering the context information.

Method	Walk 1	Walk 2	Walk 3	Average Accuracy
Trilateration	0.52 m	0.95 m	0.71 m	0.73 m
Dead Reckoning	5.61 m	2.79 m	3.96 m	4.1 m
Fusion	1.02 m	0.57 m	0.63 m	0.74 m

**Table 20:** Positioning accuracy of final position after each trial with the context information.

Method	Walk 1	Walk 2	Walk 3	Average Accuracy
Trilateration	0.37 m	0.78 m	1.00 m	0.71 m
Dead Reckoning	0.20 m	1.60 m	1.32 m	0.98 m
Fusion	0.88 m	0.50 m	1.07 m	0.82 m

**Table 21:** Summary of the obtained results.

Approach	Advantages	Disadvantages
Trilateration	moderate accuracy	additional hardware required
	constant error	jumpy position estimation
	no start position required	sensor interference
Dead Reckoning	moderate accuracy no additional hardware required	growing error
		sensor interference
		start position required
Fusion	high accuracy	additional hardware required sensor interference
	constant error	
	no start position required	
